# Achieving the optimal emergence profile: the role of soft tissue grafting and pontic site development

**DOI:** 10.1038/s41415-024-8023-2

**Published:** 2024-12-13

**Authors:** ﻿Matthew Brennand Roper, Yasmin Fields

**Affiliations:** 41415284390001 Consultant in Restorative Dentistry, Bristol Dental Hospital, Lower Maudlin Street, Bristol, BS1 2LY, UK; 41415284390002 Specialty Registrar in Restorative Dentistry, Bristol Dental Hospital, Lower Maudlin Street, Bristol, BS1 2LY, UK

## Abstract

Hard and soft tissue remodelling after tooth extraction may result in a concave profile at the subsequent edentulous ridge. This defect may result in a sub-optimal aesthetic transition zone between the soft tissue and the pontic, which may appear to sit on the ridge, rather than emanating from within the ridge, as would a natural tooth. To optimise aesthetics, pontic site augmentation (PSA) (increasing the volume at the pontic site) and pontic site development (PSD) (remodelling the tissue at the pontic site) may provide a solution.

This article discusses the role of soft tissue grafting for PSA, alongside the techniques employed for PSD. Biomaterial substitutes may be used for soft tissue grafting; although autogenous tissue remains the gold standard. Patients may benefit from biomaterial substitutes (as no donor site is required) but evidence for long-term volumetric stability within this specific scenario is limited.

Studies suggest PSD may be initiated three months post-augmentation, with minimal changes in site volume following this time point; although again, clinical data are limited. PSD can be achieved via several techniques, depending on operator and patient preference, with the ultimate goal of tissue conditioning to accept a convex fit surface that facilitates hygiene practices. PSA and PSD play key roles in the creation of a natural emergence profile at edentulous sites, leading to optimal aesthetics and cleansability.

## Key points

- Compares the types of soft tissue grafts available for pontic site augmentation.
- Discusses the stages involved in soft tissue grafting and the evidence regarding the optimal healing interval.
- Explores the different methods of pontic site development.

## Introduction

**Fig. 1:**
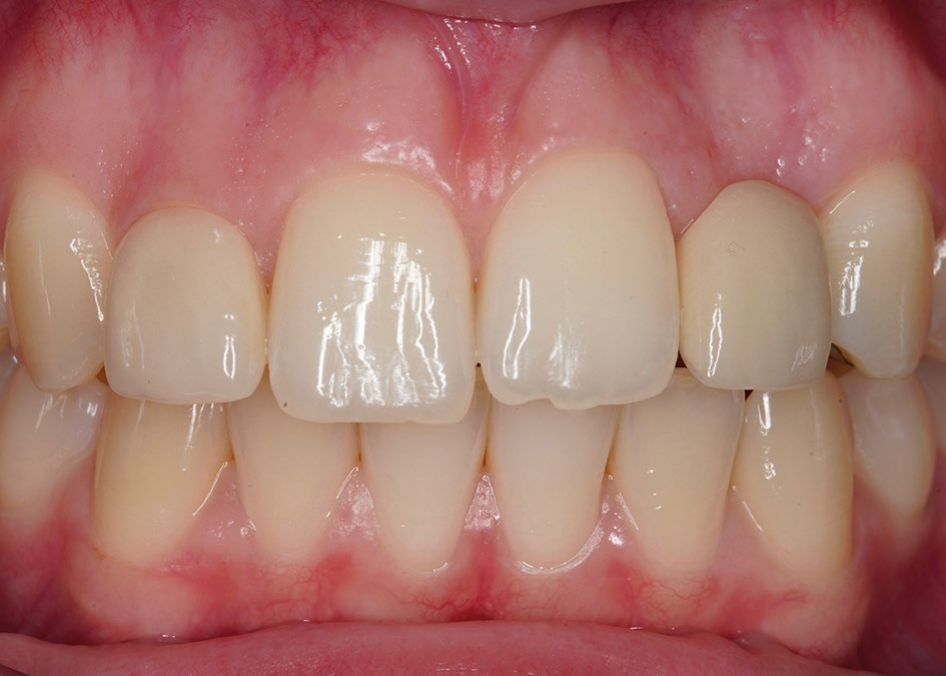
Volumetric deficiency above 22 pontic

Dimensional changes occur after tooth extraction, with one-third of the ridge width lost within the first three months and 50% at 12 months.^1^ This volumetric loss results in a concavity at the facial surface of the alveolar ridge and may detract from the provision of a natural-looking prosthesis because the emergence profile often appears to emanate from the surface of the ridge, rather than from within the ridge. When the smile line exposes the junction between the tooth and gingiva, the volumetric deficiency above the pontic creates a shadow, as this region is not illuminated in the same way as the root bulbosity of natural teeth (Fig. 1). These factors can significantly detract from the pink aesthetic score, as reported by Furhauser and modified by Belser.^2^^,^^3^ Table 1 provides a summary of the parameters assessed. To optimise aesthetics, the lost volume must be replaced via surgical intervention or prosthetic replacement.

**Table 1:** Summary of the pink aesthetic score parameters

Parameter	Score
Mesial papilla	2 = complete presence of mesial papilla 1 = incomplete presence 0 = absent
Distal papilla	2 = complete presence of distal papilla 1 = incomplete presence 0 = absent
Curvature of facial mucosa	2 = identical to natural tooth contour 1 = slightly different 0 = markedly different
Level of facial mucosa	2 = identical vertical level compared to the contralateral tooth 1= slight (≤1 mm) discrepancy 0 = major (≥1 mm) discrepancy
Presence of a convex root profile, soft tissue colour and soft tissue texture	2 = all three parameters are similar to a natural tooth 1 = two parameters are similar to a natural tooth 0 = one or none of the parameters are similar to a natural tooth
Total score (cumulative score of the five parameters above)	≥6 = clinically acceptable

Pontic site augmentation (PSA) refers to the surgical augmentation of an edentulous site. This is followed by pre-prosthetic development to optimise the topography for a convex pontic fit surface. This article describes these steps, with the aim of emulating the emergence profile of a natural tooth.

Combined hard and soft tissue grafting, techniques involving socket shields, or alveolar ridge preservation are beyond the remit of this article.

## Augmentation

When considering soft tissue augmentation, available graft materials include autogenous connective tissue, allografts, or biomaterial substitutes. Table 2 provides a brief summary of the different types of soft tissue grafts which may be considered for this procedure.

**Table 2:** Overview of the types of autogenous and biomaterial forms of grafting

Autogenous connective tissue grafts	Biomaterial substitutes
Subepithelial connective tissue graft	Xenograft (animal derived)
De-epithelialised free gingival graft	Allograft (human derived)

Autogenous connective tissue grafts can either be harvested as a subepithelial connective tissue graft (SCTG) or as a free gingival graft (FGG) which is subsequently de-epithelialised (DFGG). De-epithelialisation may be carried out either before or after harvest, depending on the operator's preferred technique. The use of a SCTG was first reported by Langer and Calagna in 1982 to develop pontic sites.^4^ However, to the authors' knowledge, there is still no available literature comparing SCTGs to DFGGs for pontic site augmentation, with the majority of available evidence referencing the SCTG. Autogenous connective tissues can be harvested from two sites, depending on the quality and quantity of tissue required. Generally, the middle third of the hard palate is used, with the possibility to extend anteriorly depending on the size of the graft required. Alternatively, grafts can be harvested from the tuberosity, which can deliver significant quantities of dense connective tissue, depending on the individual anatomy of the patient.

**Fig. 2:**
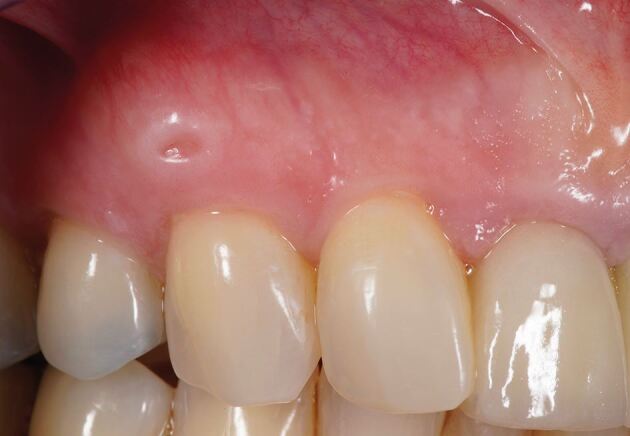
Fenestration of the DFGG epithelium through the overlying mucosa

The phenotype of the graft harvested is important to consider. Grafts taken from the superficial layers, such as the DFGG or the tuberosity (which is predominantly composed of dense connective tissue), may increase in volume over time^5^ and can create aesthetic complications if the underlying regenerated epithelium is not fully de-epithelialised (Fig. 2). Patients with a thin phenotype may have limited submucosal tissue and harvesting a SCTG may not be feasible, favouring the DFGG technique. Tissue sounding may provide some insight peri-operatively as to the viability of harvesting an SCTG.

Alternative substitute biomaterials may remove the need for an autogenous donor site. These materials are derived from either animal origin (xenografts) or are human-derived allografts. They are comprised of either native collagen matrices or crosslinked collagen matrices; this decision is based on the desired substitution or resorption traits, with the fundamental requirement being that the desired volume augmentation is maintained. Research in an animal model identified that the use of a crosslinked collagen matrix resulted in a greater and more stable ridge width over time compared with control groups using native collagen matrices.^6^ These biomaterials have a range of physical characteristics, from a sponge-like consistency (Geistlich Fibro-Gide) to a form more similar to a DFGG, such as BioHorizons' NovoMatrix (acellular dermal matrix). There is a limited body of evidence available reporting on the long-term outcomes of these collagen matrices when compared to that of autogenous connective tissue grafts, which remain the gold standard.^7^

When the defect is significant, soft tissue grafting alone may not adequately augment the site to full contour and consideration may be given to combined hard and soft tissue grafting. The reader is directed towards bone augmentation texts for further information on these procedures.

## Surgical intervention

**Fig. 3:**
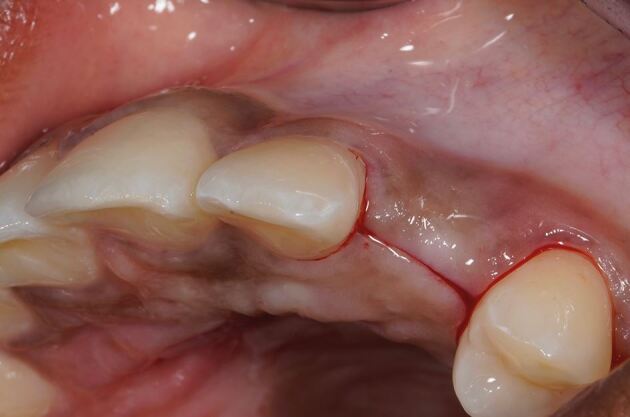
Envelope incision

**Fig. 4:**
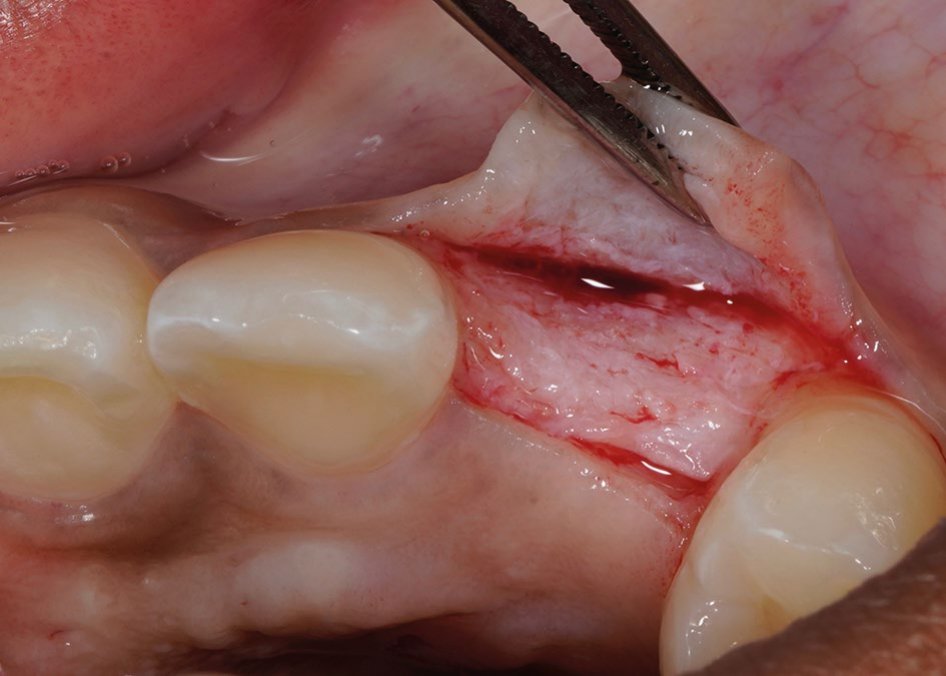
Split thickness flap

**Fig. 5:**
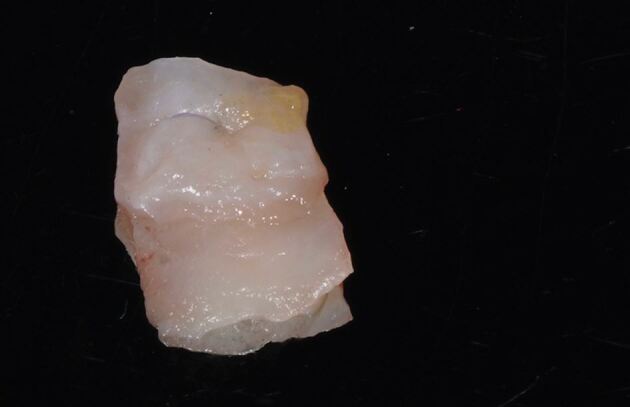
DFGG harvested from the palate and folded to double the thickness

The recipient graft site (pontic site) is accessed using an envelope-style incision (Fig. 3) or a tunnel preparation. Both deliver a split thickness flap (Fig. 4) or pouch to accept the graft. This leaves the periosteum intact and provides a vascularised bed for the recipient graft, while the superficial connective tissue and epithelium is freed from the underlying attached mucosa as a split thickness layer. This allows for tension-free closure over the inter-positional graft. Initial graft survival depends on plasmatic circulation for nourishment while angiogenesis takes place. The envelope flap improves surgical access to appropriately position the graft, whilst a pouch created via tunnelling reduces the size of the surgical wound at the ridge crest. This however increases the complexity of the procedure and reduces surgical access. Figure 5 shows a DFGG harvested from the palate and folded to double the thickness, providing a compression resistant matrix for augmentation of the buccal aspect.

**Fig. 6:**
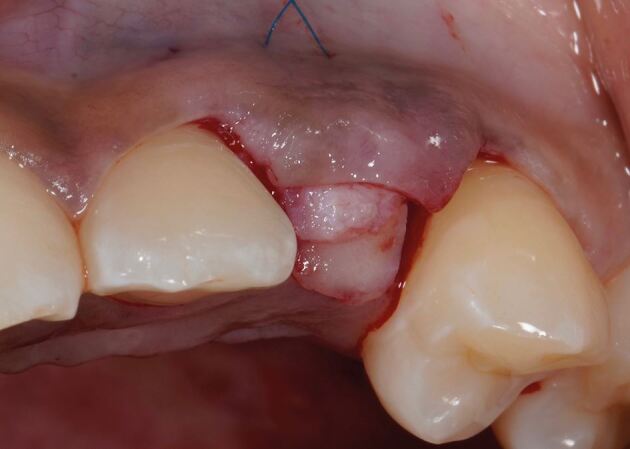
Introduction of the graft into the pontic site with a securing suture through the buccal flap

**Fig. 7:**
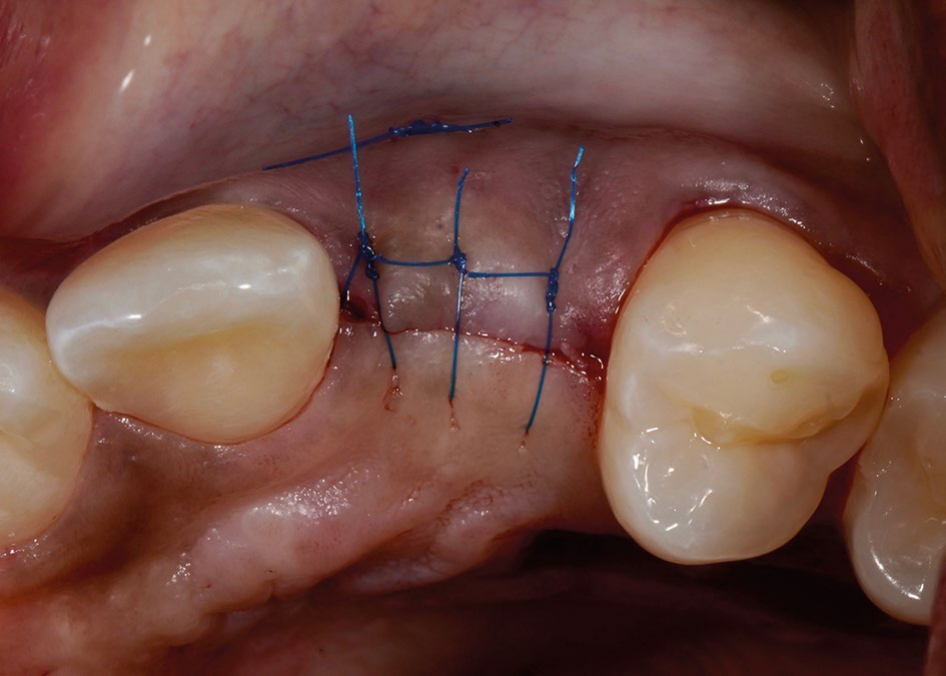
Immediate postoperative image; 6-0 proline sutures have been used to close the incision

**Fig. 8:**
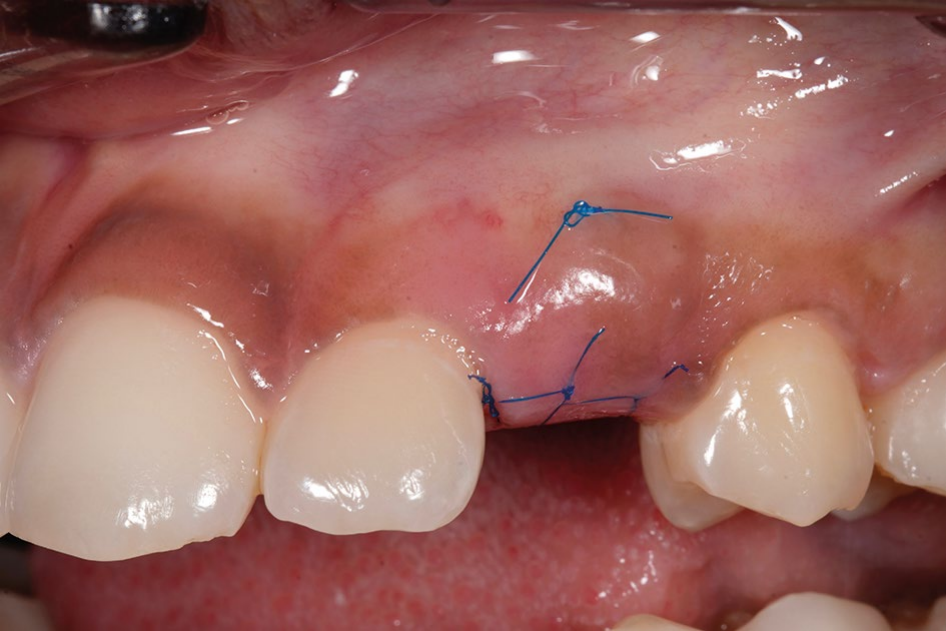
Healing at two weeks, before suture removal

**Fig. 9:**
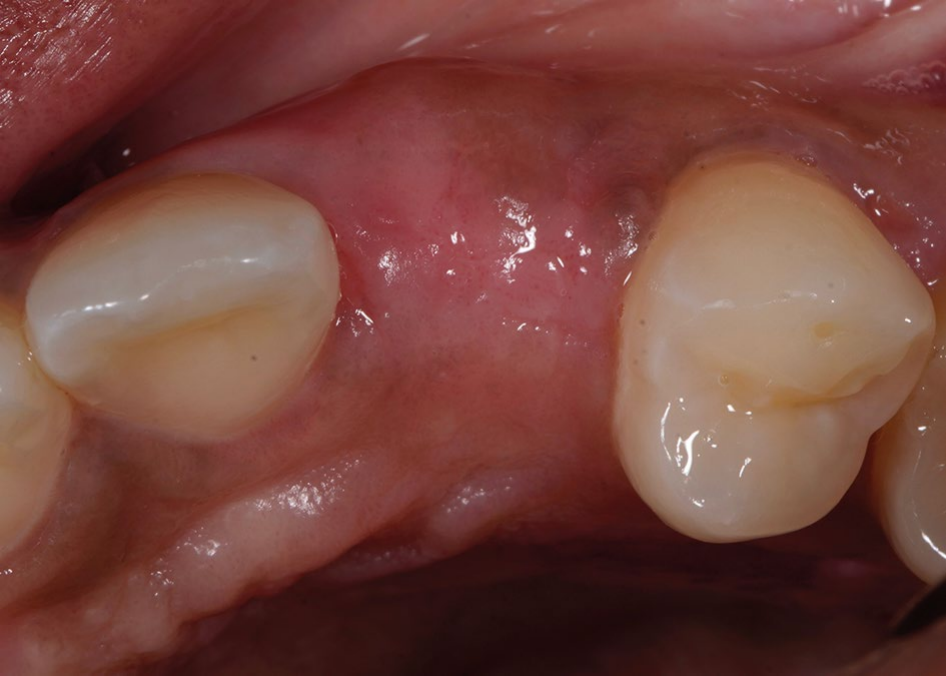
Bucco-lingual volumetric increase at one month

The connective tissue graft or substitute collagen matrix can be secured onto the prepared bed via one of two techniques. The first secures the graft material onto the recipient bed itself with a mattress sling suture, while the second secures the graft to the buccal flap, which is further stabilised upon closure. The choice depends on the surgical access to the recipient bed, the remaining thickness of periosteum to suture to and operator preference. The key parameter is to ensure the graft material is placed in the correct three-dimensional position to augment the buccal aspect. Figure 6 shows the introduction of graft into the pontic site, correctly positioned, with a securing suture through the buccal flap. Figure 7 shows the immediate post-operative image using 6-0 proline sutures to close the incision. Care is taken to ensure this is tension-free. Figure 8 shows healing at two weeks, before suture removal. Figure 9 demonstrates the bucco-lingual volumetric increase at one month.

## Donor site harvest

If an autogenous connective tissue graft is chosen, a second surgical site is required. These donor sites are subject to the same risks as the recipient graft site.

**Fig. 10:**
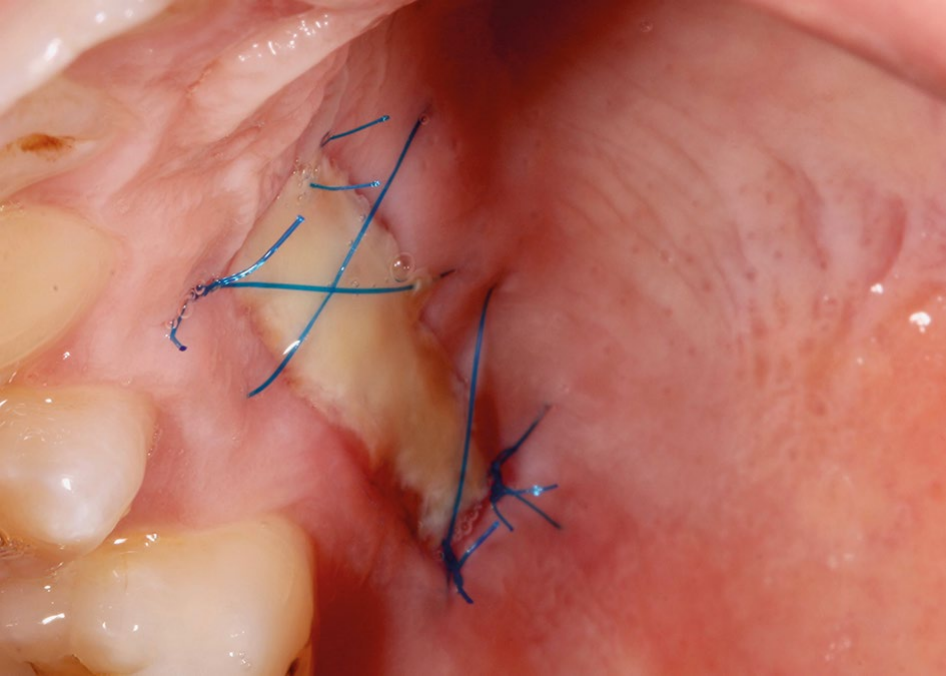
Early healing with a native collagen matrix becoming integrated into the donor site

If a DFGG is harvested, the level at which the graft is separated from the base can influence the degree of haemorrhage. If the graft is harvested just superficial to the glandular mucosa, often, intra-operative bleeding is significantly reduced, as this region is comprised of dense connective tissue. The anatomy of the palate is described elsewhere, but in the main, the risk of damage to larger vessels in the area (such as the greater palatine artery) is significantly reduced if the greatest extension towards the palatal midline is 10 mm from the palatal aspect of the cemento-enamel junction, with the graft harvested anterior to the mesial aspect of the first maxillary molar palatal root. After DFGG harvest, the authors' preference is use of an Essix-style healing plate, which covers the palate without further surgical intervention. Other techniques involve the use of biomaterial matrices to cover the de-epithelialised site, stabilised via sutures. This management may however, cause additional bleeding and the sutures require removal at a later date. Figure 10 illustrates early healing with a native collagen matrix becoming integrated into the donor site. This adds time and cost to the procedure.

**Fig. 11:**
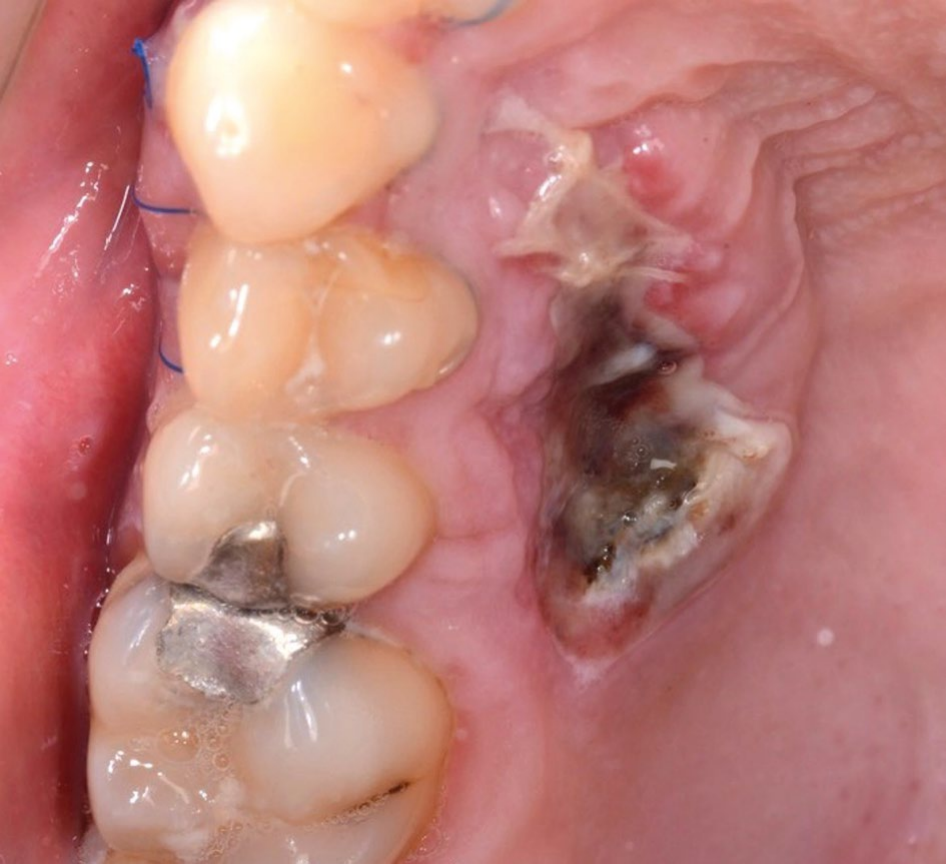
Necrosis at the connective tissue graft donor site

If a SCTG is harvested, the access incision is closed with sutures. Care must be taken to ensure that the superficial layer is not left too thin, as necrosis can occur (Fig. 11). Patient-reported outcomes comparing visual analogue pain scores between patient groups receiving SCTGs and collagen matrices identified a greater need for post-operative analgesia when autogenous grafts were used compared to biomaterials substitutes.^8^ No statistically significant differences were demonstrated between patients undergoing a SCTG compared to a DFGG, but pain increased when there was necrosis of the overlying mucosa.^9^

In the post-surgical phase, the Essix-style healing plate (which extends over the palate and onto the incisal edges for retention) is worn continuously for the first 24 hours, then during the day for the first week to prevent trauma when eating and speaking. The appliance is to be removed for cleaning after meals and disinfected twice a day using a chlorhexidine product. For most patients, missing an anterior tooth will be socially unacceptable, so the healing plate may be constructed with a replacement tooth, or as a Hawley-style retainer.

Suture removal takes place at two weeks and the patient is reviewed at three months to begin pontic site development.

## Graft stability

Pontic site development (PSD) transforms the soft tissue topography from a convex ridge to a concavity with a scalloped margin, ready to accept an ovate pontic. This design allows for cleansability of the underside of the prosthesis, while delivering an emergence profile projecting from within the soft tissue to optimise the aesthetic outcome.

When planning the restorative phase of treatment, consideration should be given to the timing of prosthesis placement following soft tissue augmentation, as soft tissue stability is desired. An investigation examining single-site pontic development using connective tissue grafts identified significant volumetric changes up to three months from baseline, but non-significant changes in volume between three and six months.^10^ Longer-term studies have shown that soft tissue volumes at pontic sites, with and without a SCTG, were stable at five and ten years with no statistical difference between the groups.^11^^,^^12^ When comparing porcine collagen matrices to a STCG, an animal model identified significant volumetric changes within the first month but no statistical significance in terms of soft tissue volumes between the two groups at ten months.^13^ Histological examination also reported comparable quality of the augmented connective tissues between the two groups at ten months.^14^

Although limited, the available evidence suggests that the final reconstruction may be considered three months post-augmentation at the earliest, with soft tissue stability expected over the longer term.

**Fig. 12:**
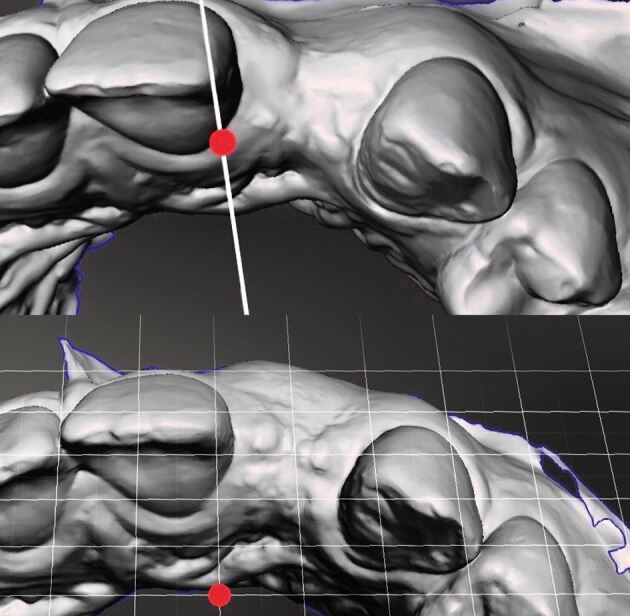
Digitised images of the clinical situation pre- and post-augmentation as STL files

Digital technologies may also assist clinicians in choosing the appropriate time for reconstruction via superimposed images captured from intra-oral scanners (IOS). Volumetric changes can be mapped, and when stable, reconstruction considered. Figure 12 shows digitised images of the clinical situation pre- and post-augmentation as steriolithic tessellated language (STL) files. These technologies allow clinicians to deliver personalised, patient-specific treatment strategies. Further information regarding the use of IOS devices can be found elsewhere.^15^

## Pontic site development techniques

Pontic site development may be carried out before impressions for the definitive prosthesis, or at the time of fitting the definitive restoration.

**Fig. 13:**
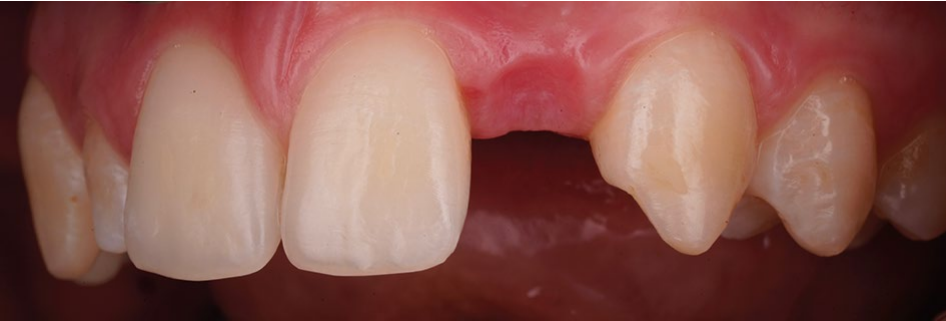
Thin gingival phenotype

**Fig. 14:**
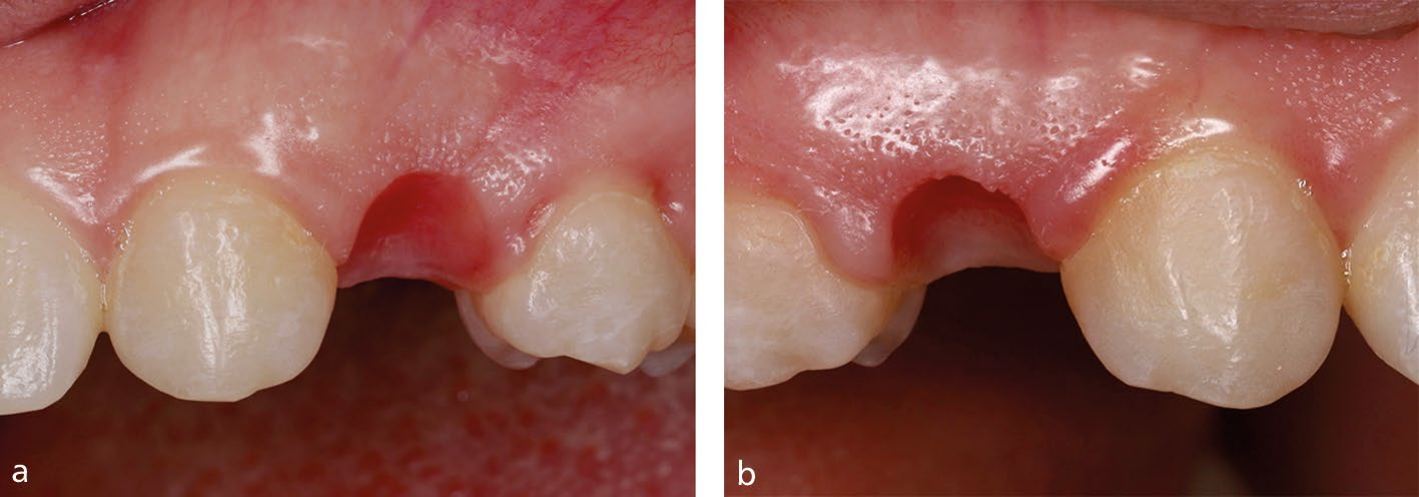
a, b) Thick gingival phenotype

Pontic site development before restoration can be conducted either with a removable provisional prosthesis or a fixed provisional prosthesis. Essix-style or Hawley-style retainers are commonly used, with materials such as composite resin (flowable or conventional), acrylic resins, or bis-acrylic composites chosen to augment the fitting surface of the pontic. When a removable prosthesis is used, immediate delivery of an ideally shaped ovate pontic may prevent full seating; therefore, smaller staged additions may be required to allow tissue compression without significant prosthesis displacement. Prosthesis use in function will steadily compress the tissue and develop the pontic site. This may take several weeks to complete and may be inconvenient for the patient but generally prevents the need for local anaesthetic and a surgical procedure. The patient's phenotype may dictate the level of soft tissue compression available, with thin phenotypes limiting tissue compression, as shown in Figure 13, when compared to thicker phenotypes, as shown in Figure 14.

**Fig. 15:**
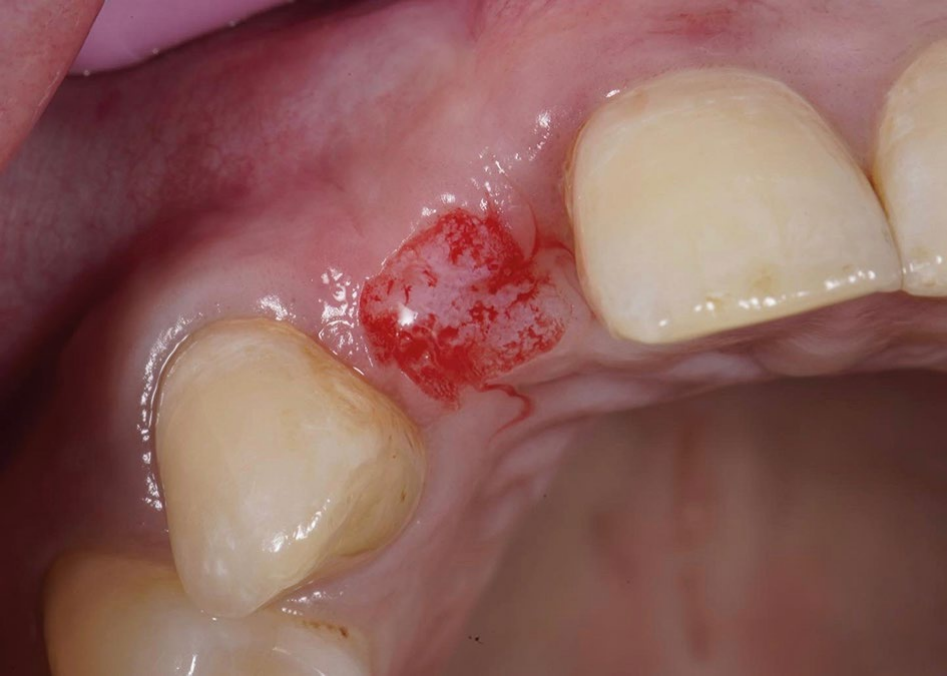
Pontic site development which has been carried out with a round diamond bur

The surgical approach to immediate pontic site development is carried out under local anaesthetic. Techniques include the use of a tissue removal bur, round diamond bur (Fig. 15), electrosurgery, or a tissue punch to remove the desired volume of soft tissue. The provisional prosthesis with the desired fit surface shape is delivered and will maintain the newly created concavity. The site is subsequently left to heal over a period of 2-4 weeks, with any subsequent impressions reproducing the customised soft tissue profile.

**Fig. 16:**
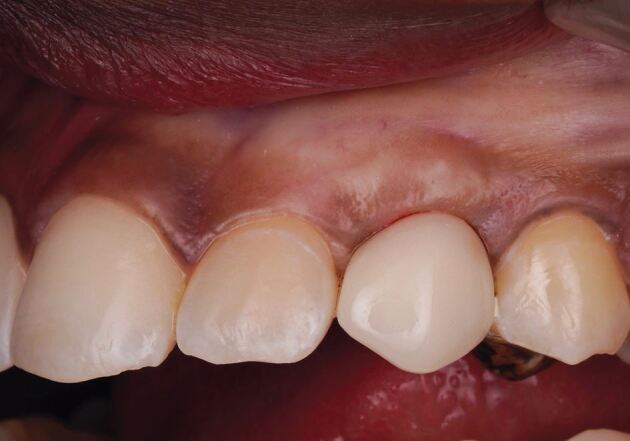
The final bridge at the fit appointment, immediately after surgical pontic site development to match the laboratory-constructed fit surface

The laboratory-based strategy develops the pontic site on the cast when the definitive prosthesis is made, with soft tissue modification at the time of fit. This pathway benefits the patient in terms of immediacy of reconstruction. This strategy carries two challenges. Firstly, with the laboratory estimating the degree of soft tissue removal required and secondly, with respect to haemostatic control at cementation, which is imperative to prevent contamination at the time of delivery. Figure 16 illustrates the final bridge at the fit appointment, immediately after surgical pontic site development to match the laboratory-constructed fit surface.

## Conclusion

PSA and PSD are often required to modify the topography of an edentulous ridge to accept an ovate pontic with an optimal emergence profile. This gives the illusion of a prosthesis emanating from the mucosa, mimicking a natural tooth, while delivering optimal anatomy for patient-conducted oral hygiene.

Autogenous connective tissue or biomaterial substitutes may be used for soft tissue grafting. Autogenous grafting remains gold standard; however, it involves a second surgical site that requires post-operative management and increases surgical morbidity.

Pontic site development may be conducted as early as three months post-augmentation, with some evidence to support stability of the soft tissues in the long-term. Following augmentation, prosthetic or surgical interventions may be used to achieve adequate PSD, with the chosen technique related to operator and patient preference and clinical situation. PSA and PSD play a key role in delivering natural aesthetics and meeting patient expectations. However, limitations exist where defects cannot be managed by soft tissue grafting alone and hard tissue grafting may also be required.

Further research is still needed to compare SCTG to DFGG for pontic site development and to assess the long-term outcomes of collagen matrices compared to autogenous connective tissue grafts.
